# Supplementary material for: Complex‐centric proteome profiling by SEC‐SWATH‐MS
Source: Mol Syst Biol. 2019 Jan 14;15(1):e8438. doi: 10.15252/msb.20188438 (PMC6346213; doi:10.15252/msb.20188438)
Supplement: Supplementary file 8 — Dataset EV7 [file MSB-15-e8438-s008.zip › feature_plots_string/P82914.pdf]

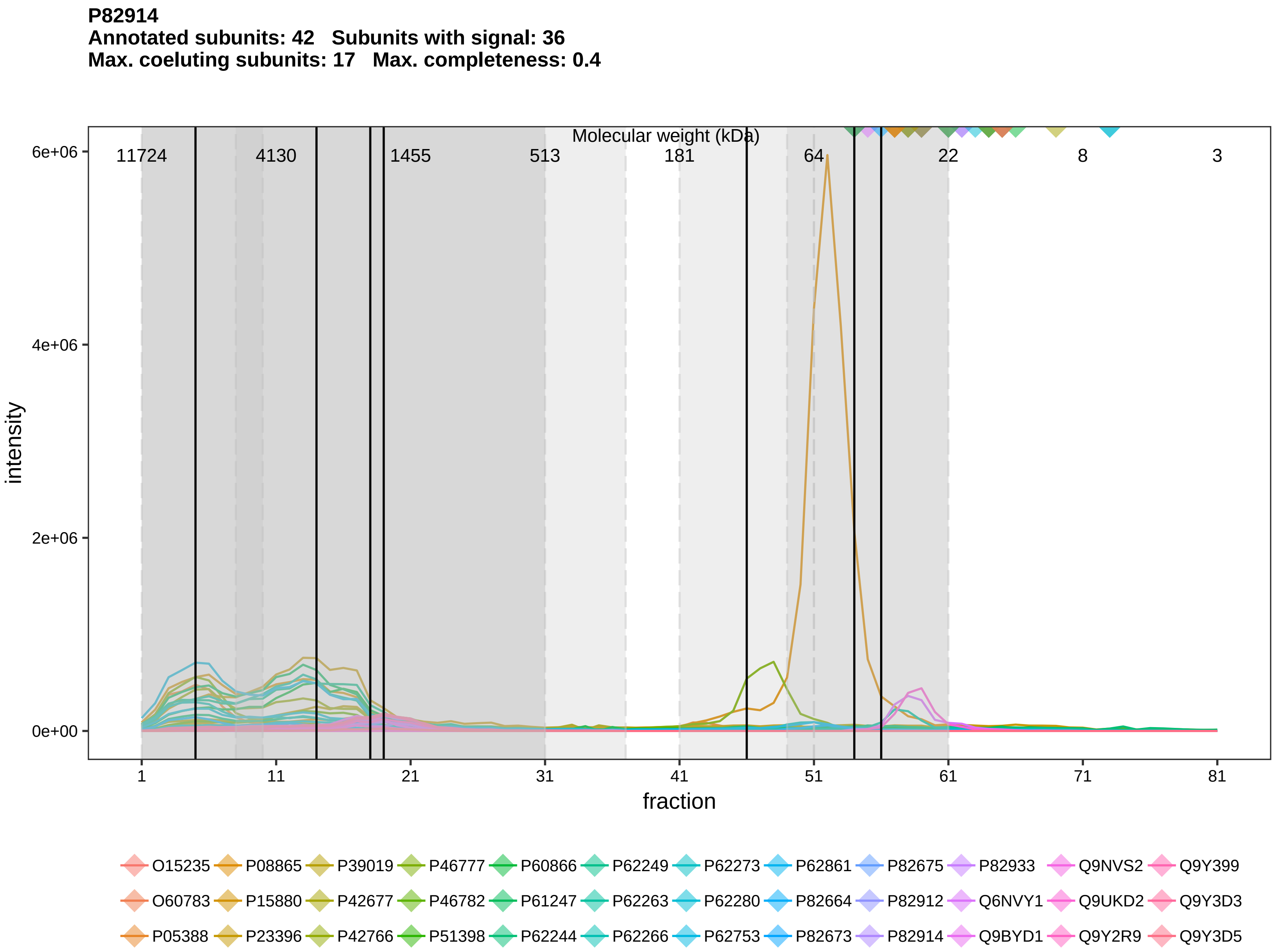

P82914
Annotated subunits: 42   Subunits with signal: 36
Max. coeluting subunits: 17   Max. completeness: 0.4
Molecular weight (kDa)
11724
4130
1455
513
181
64
22
8
3
6e+06
4e+06
2e+06
0e+00
intensity
1
11
21
31
41
51
61
71
81
fraction
O15235
P08865
P39019
P46777
P60866
P62249
P62273
P62861
P82675
P82933
Q9NVS2
Q9Y399
O60783
P15880
P42677
P46782
P61247
P62263
P62280
P82664
P82912
Q6NVY1
Q9UKD2
Q9Y3D3
P05388
P23396
P42766
P51398
P62244
P62266
P62753
P82673
P82914
Q9BYD1
Q9Y2R9
Q9Y3D5
